# Altered staining patterns and expression level of Engrailed-2 in benign prostatic hyperplasia and prostate Cancer predict prostatic disease progression

**DOI:** 10.1186/s12885-020-07049-z

**Published:** 2020-06-15

**Authors:** Qi Li, Yibo Shi, Rigai Sa, Jun Hao, Jinhao Hu, Mulun Xiao, Chaoliang Wang, Liang Yan, Baoping Qiao, Guoxun Chen

**Affiliations:** 1 Department of Urology, The First Affiliated Hospital of Zhengzhou University, Zhengzhou, Henan China; 2 Beijing Gegen biotechnology co., LTD, Beijing, China; 3 Interdisciplinary life science, Purdue University, West Lafayette, IN USA; 4 Department of Nutrition, University of Tennessee at Knoxville, Knoxville, TN USA

**Keywords:** Prostate cancer, Benign prostatic hyperplasia, Engrailed-2, Immunohistochemical staining

## Abstract

**Background:**

Prostate cancer (PC), a common malignant tumor, is the second-leading cause of cancer death among American men. Its successful treatment greatly relies on the early diagnose. Engrailed-2 (EN2) has been confirmed being existed with a high level in the urine of PC patients. In this study, to explore the application of EN2 in PC, we detected the immunohistochemical staining difference and EN2 expression level between benign prostatic hyperplasia (BPH) and PC.

**Methods:**

We developed a monoclonal antibody against the helix 3 in EN2 and confirmed its specificity with Western blotting (WB) and immunofluorescence detecting the subcellular localization of endogenous and exogenous EN2 in three PC cell lines (LNCap, PC3, and DU145). We conducted immunohistochemical staining using this homemade antibody, and RT-PCR to detect the expression of EN2 in 25 PC and 25 BPH cases, and analyzed the correlation of EN2 expression and PC clinical staging.

**Results:**

The results of WB and immunofluorescence showed our homemade EN2 monoclonal antibody could specifically bind endogenous and exogenous EN2 protein in three different PC cell lines. Endogenous EN2 was generally expressed in the cytoplasm and exogenous EN2 mostly existed in the nucleus of these cell lines. Immunohistochemical staining in PC had extremely stronger signals than that in BPH, suggesting a higher EN2 expression level in PC, which was confirmed by RT-PCR. Interestingly, the stained areas in BPH tissues were mainly in nucleus and cytoplasm, while in PC tissues were mainly on cytomembrane. Moreover, the expression level of EN2 was positively correlated with the PC clinical staging.

**Conclusion:**

Using our homemade EN2 antibody, we have found different staining patterns and expression level of EN2 in BPH and PC,which may be helpful to predict prostatic disease progression.

## Background

Prostate cancer (PC) is the most common cancer diagnosed among males in the US and the second cause of cancer death in men [1]. The extremely high morbidity and mortality make PC one of the most serious threats to men’s health [2]. Nowadays, the PC treatments only rely on surgery or radiotherapy when the cancer is still in the localized stage [3, 4]. Thus the survival rate would be commonly improved if PC could be diagnosed in the early stage. Although there has been vast of progress in understanding PC pathobiology, there is no approved test for the diagnosis and monitoring of PC except for prostate-specific antigen (PSA) test. However, PSA is not used widely as a diagnostic marker for PC due to its low specificity and sensitivity [5].

Benign prostatic hyperplasia (BPH) is a kind of prostatic nonmalignant hyperplasia and may cause serious symptoms such as lower urinary tract symptoms (LUTS) which undermines patients’ life quality [6]. Meta-analyses data shows BPH is associated with an increased incidence of prostate cancer and these two prostatic diseases have certain similar traits such as androgen-dependent growth or response to hormonal therapy, which lead to overtreatment or delayed treatment of the diseases [6, 7]. To judge the state of prostatic disease better, new biomarkers are needed to show some meaningful clues in the process of prostate diseases.

Recently, studies have shown that the dysregulation of homeobox (HOX) gene family occurs in many cancers, including solid and hematological malignancies [8]. Engrailed-2 (EN2), a member of the HOX gene family, has been found to overexpress in various kinds of cancers like PC, breast cancer and bladder cancer, and play important roles in oncogenesis [8, 9]. It has been reported that positive detection of EN2 in patients’ urine with ELISA was predictive of PC with high sensitivity and specificity (66 and 88.2% respectively) [10]. Moreover, a strong positive correlation was shown not only between pre-surgical level of urinary EN2 and the volume of cancerous tissues removed in prostatectomy, but also between EN2 levels and tumor stages [11]. Since EN2 can be detected in urine after prostate carcinogenesis, intracellular EN2 may change expression form because normal prostate tissue and hypertrophic prostatic cells do not secrete EN2 [10, 11]. All these results suggest a potential for EN2 as a candidate biomarker in early detection of PC or the differential diagnosis for PC and BPH.

Structurely, the full length of EN2 protein has 333aa, including three alpha helices, with the helix 1 and 2 at the N end binding DNA, and helix 3 at C end, mainly mediating the exocrine and internalization of EN2 protein [12, 13]. We produced a monoclonal antibody targeting Helix 3 in EN2, and conducted immunohistochemical staining with this homemade antibody to detect EN2 expression patterns in 25 BPH and 25 PC cases. The EN2 expression levels of these cases were confirmed by RT-PCR. We also analyzed the EN2 immunohistochemical scores, the clinical indicators, and their correlation in PC cases and found the expression level of EN2 was positively correlated with PC clinical progress.

## Methods

### Ethics statement

This study involving human participants was approved by the ethics committee of The First Affiliated Hospital of Zhengzhou University. The audit number of ethics committee was 2019-KY-185. Written consent was obtained from all human participants. Research was carried out according to the principles expressed in the Declaration of Helsinki.

### Patients and samples

Clinical samples and patient records corresponding to 50 consecutive patients diagnosed as PC or BPH at the Urology Department of The First Affiliated Hospital of Zhengzhou University between January 2017 and October 2018 were examined. The inclusion criteria were as following: (1) Only patients who came to our hospital for the first time for examination and diagnosis were collected; (2) No other systemic tumors, severe infection and trauma; (3) 1 week before the examination of serum PSA, there was no indwelling catheterization, cystoscopy, digital rectal examination and other operations affecting the result of serum PSA and inflammatory indicators; (4) No endocrine treatment; (5) No coagulation dysfunction, serious cardiovascular and cerebrovascular diseases. Patients underwent Laparoscopic radical prostatectomy and cancer or hyperplasia samples were split in half immediately after resection. One half was embedded with paraffin, and the other half was immediately snap-frozen in liquid nitrogen for RT-PCR analyses. All the samples used were confirmed by a pathologist.

### Cell culture and experimental reagents

Human PC cell lines, LNCaP clone FGC cells (LNCaP) (ATCC CRL-1740 cells), DU145 (ATCC HTB-81 cells) and PC3 (ATCC CRL-1435 cells), and human embryonic kidney cell line (293 T) (ATCC CRL-11268 cells) were bought from ATCC in January 2017. All these cells were authenticated with short tandem repeat (STR) analysis by ATCC. Mycoplasma contamination test was done with PCR Detection Kit for Mycoplasma Contamination (#Myco-P-20, Shanghai Yanxi biotechnology co., LTD, China). LNCap cells were cultured with Roswell Park Memorial Institute (RPMI) 1640 medium (#11875093, Gibco, USA), DU145 cells were cultured with Dulbecco’s Modified Eagle’s Medium (DMEM) (#11995040, Gibco, USA), PC3 cells were cultured with DMEM/F12 (#11330057, Gibco, USA), and 293 T cells were cultured with DMEM (#11995040, Gibco, USA). The culture medium was supplemented with 10% fetal bovine serum (#16140089, Gibco, USA), 100 U/ml penicillin and 100 μg/ml streptomycin (#15070063, Gibco, USA) when used and cells were incubated at 37 °C in a humidified incubator with 5% CO2. Ethical clearance to use these human cell lines was obtained from the Ethics Committee for Research Involving Human Subjects, The First Affiliated Hospital of Zhengzhou University. [No. 2019-KY-185].

### Preparation of monoclonal antibodies against Engrailed-2

C-terminal 114aa of EN2 was selected according to EN2 mRNA sequence (NCBI Reference Sequence: NM_001427.3) to prepare the EN2 monoclonal antibodies. Briefly, the gene encoding C-terminal 114aa of EN2 was synthesized in Sangon Biotech (Shanghai, China) Co., Ltd. A hexahistidine tag was added to the carboxyl terminus to aid in the detection and purification of the final protein product. The resulting gene was cloned into the expression vector pET30a and transformed into *E. coli* strain *BL21(λDE3)*. Single bacterial colony was inoculated into LB medium and grown at 37 °C to OD600 of 0.6. Expression of the recombinant EN2C protein was induced with 0.1 mM isopropyl β-D-1-thiogalactopyranoside at 37 °C for 4 h. The culture mixture was centrifuged at 2000×g for 15 min, and supernatant was collected. Soluble EN2 C-terminal 114aa was purified by Ni + affinity column and used to immunize Balb/c mice. The spleenocytes from the immunized Balb/c mice were obtained and fused with Balb/c mouse myeloma cells by hybridoma technique, and monoclonal antibodies were obtained by screening. The affinity and specificity of EN2 monoclonal antibodies were identified through ELISA, WB, immunofluorescence and immunohistochemistry.

### Western blotting

EN2 protein or cell total protein were run WB to identify specificity of EN2 monoclonal antibody. Three prostate cancer cell lines, PC3, DU145, LNcap and transfected 293 T cell were used. Cells grown in a 100 mm cell culture dish were rinsed with PBS buffer prior to harvesting. Total proteins were extracted with Cell Culture Lysis 1 × Reagent (#53711–5399, Promega Inc., USA) according to the product instruction and incubated on ice for 10 min. The cell lysate was separated by centrifugation at 12,000 x g for 2 min. The protein concentation was measured by BCA Protein Assay Reagent (#PC0020, Solarbio Inc., China). For SDS-PAGE, a total of 20 μg of protein was loaded per well. Polyvinylidenedifluoride (PVDF) membranes (Roche Diagnostics, USA) were used for the transfer process. For WB, PVDF membranes after protein transfer were incubated in 5% skimmed milk blocking buffer for 1 h, followed by washing 3 times with PBST (buffered saline plus 0.05% Tween 20). EN2 was detected using the monoclonal antibody and horseradish peroxidase-conjugated goat anti-mouse IgG secondary antibody (1:10,000; #ZB-2305; ZSGB-BIO Inc., China).

### Immunofluorescence

The recombinant pcDNA3.1-EN2-Red fluorescent protein (RFP) was transfected into 293 T or prostate cancer cell lines PC-3, DU-145 or LNCap, using PEI (polyethylenimine linear, #23966, Polysciences Inc., USA). After incubating at 37 °C, 5% CO_2_ for 6 h, the culture medium was replaced with DMEM complete medium and cultured for another 24 h. The culture medium was aspirated, and cells were fixed by incubating with 4% paraformaldehyde (#P1110,Solarbio Inc., China) for 15 min at room temperature. After washing twice in PBS buffer, freshly prepared 0.2% Triton X-100 (#T8200, Solarbio Inc., China) was added and incubated for 10 min at room temperature. Block with 5% BSA for 30 min. The monoclonal antibody of EN2 was added and incubated at 37 °C for 2 h. The Goat anti-mouse IgG/FITC (#SF131, Solarbio Inc., China) was added and incubated for 35 min at 37 °C in the dark. After washing twice with PBS, it was observed under a fluorescence microscope.

### Immunohistochemical staining of EN2 in PC or BPH tissues

Briefly, all paraffin embed PC tissues were cut into 3 μm sections. The slides were deparaffinized by heating at 60 °C and then immersed in xylene and rehydrated. The sections were boiled in 1 mM EDTA buffer solution (pH 9.0) for 20 min in a pressure cooker. Subsequently, endogenous peroxidase activity was quenched by immersing the samples in 3% hydrogen peroxide for 10 min. Each section was blocked in Tris-buffered saline with Tween20/5% normal goat serum (#ZLI-9022, ZSGB-Bio Inc., China) for 1 h at room temperature to block nonspecific binding. Then the sections were incubated with anti–EN2 monoclonal antibody at 37 °C for 60 min. Subsequently, a secondary biotinylated horse anti-mouse IgG solution (#ZB-2020, ZSGB-Bio Inc., China) and an avidin-biotin peroxidase reagent (#SPN9002, ZSGB-Bio Inc., China) were added onto the slides. The negative control sample was treated identically but with the isotype antibody. The color reaction was visualized by incubating with DAB solution (#ZLI-9017, ZSGB-Bio Inc., China) for 5 min. After washed thoroughly, the slides were placed in hematoxylin for redyeing. After dehydration with xylene and ethanol, the slides were sealed with neutral gum. For HE staining, the slides were placed in xylene and ethanol solution for dewaxing and hydration. After staining with hematoxylin for 5 min, the slides were rinsed for 10 min, and stained with 0.5% eosin aqueous solution for 1 min. After dehydration with xylene and ethanol, the slides were sealed with neutral gum.

### Evaluation of Immunohistochemical staining

Images were captured by a fluorescence microscope (Olympus DP74) and analyzed with the assistance of a histopathologist. The observer was blinded to the clinical diagnosis of the tissues at the time of assessment. A total of 100 cells were counted in 10 random fields (with × 400 objectives) and the percentage of positive cells was calculated. The semi-quantitative immunoreaction scoring system was evaluated based on the percentage of positive cells and the stain intensity. Regarding stain intensity, negative staining was defined as 0, mild positive was defined as 1, moderate positive as 2 and strong positive as 3. The scores of immunopositive cells were defined as follows: < 5% immunopositive cells was defined as 0 (negative); 5–25% immunopositive cells as 1 (mild); 25–75% immunopositive cells as 2 (moderate); and > 75% immunopositive cells as 3 (strong). The immunohistochemical score of each section is the sum of the stain intensity and positive cell scores.

### RT-qPCR

The EN2 gene expression was measured by reverse transcription quantitative polymerase chain reaction (RT-qPCR). The reverse transcription reaction was performed according to the manufacturer’s instructions by PrimeScript™ RT reagent Kit (#RR047A, TAKARA, Japan) and the qPCR reactions were performed at 94 °C for 5 min, 94 °C for 20 s, 55 °C for 20 s and 72 °C 15 s for 30 cycles, followed by 72 °C for 5 min using 7500 Fast Real-Time PCR System (Applied Biosystems, ThermoFisher Inc., USA). The sequences of the primers are presented in Table 1.

**Table 1:** qPCR primers

Targets	F	R
EGFR	ACGGGGTGACTGTTTGGGAGTT	ACTTTGGGCGACTATCTGCGTCT
VEGF	GGCAGAAGGAGGAGGGCAGAAT	CATCGCATCAGGGGCACACA
EN2	CTACTGTACGCGCTACTCGG	CCCGTGGCCTTCTTGATCTT
mTOR	TGGACACCAACAAGGACGAC	GTCCCACTGACCTAAACCCC
pTEN	TGGGGAAGTAAGGACCAGAGACAAAA	TGGCAGACCACAAACTGAGGATTG
GAPDH	TCGGAGTCAACGGATTTGGT	TTCCCGTTCTCAGCCTTGAC

The relative transcription level was presented as 2^-△△Ct^ of each target in PC tissues relative to BPH tissues. For the first step, we subtracted the transcription level of each target in BPH tissues from the corresponding target level in PC tissues, and got a value asΔCt. The 2^-△△Ct^ value was then be calculated at the second step. In BPH tissues, the relative transcription level of each target was obtained by subtracting the transcription level of an internal reference (GAPDH) from the level of each target in BPH tissues to getΔCt, and the relative transcription levels, represented as 2^-△△Ct^, was then be calculated at the second step.

### Statistics and methods

Data were expressed as mean ± standard deviation (SD). Data that follow the normal distribution were compared using the t-test, otherwise Mann Whitney U test was used. Counting data was expressed as composition ratio or rate (%), and comparison was made by chi-square test. EN2 immunohistochemical scores in 25 PC and 25 BPH cases were analyzed using Fisher’s Exact Test. The correlation between EN2 immunohistochemical score and clinical indicators was analyzed using spearman rank correlation. Data analysis was performed using SPSS 23 software. *P* < 0.05 was considered statistically significant.

## Results

### Homemade monoclonal antibody showed EN2 specificity

**Fig. 1:**
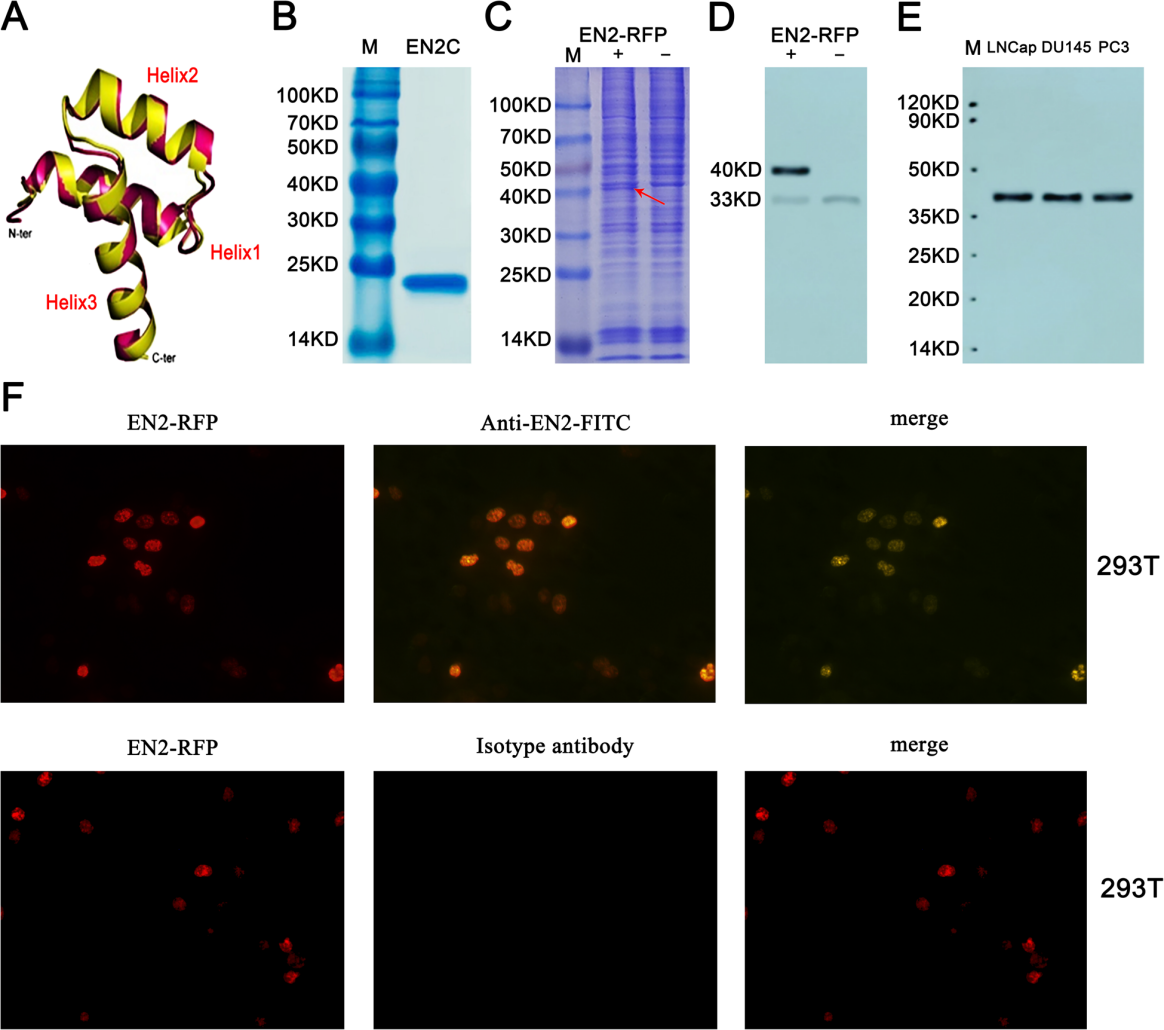
**a**. Simulation structure of EN2. **b**. C-terminal of EN2 used as immunogen. **c**. Total proteins of 293 T transfected with or without EN2-RFP-expressing plasmid. Lane M was the protein marker, lane “EN2-RFP+” was 293 T cell transfected with EN2-RFP, as the arrow points out, the band was the EN2-RFP fusion protein. Lane “EN2-RFP-” was the total 293 T cell protein. **d**. Western blot analysis of extracts from 293 T cells with or without transfection of EN2-RFP by homemade EN2 monoclonal antibody. The lane on the left was 293 T cells with EN2-RFP fusion protein (+) while the lane on the right was 293 T cells without EN2-RFP fusion protein (−). The molecular weight of EN2-RFP fusion protein was 40 KDa while the molecular weight of endogenous EN2 was 33 KDa. **e**. WB of EN2 in LNCap, DU145 and PC3 cell lines with homemade EN2 monoclonal antibody. Total cell proteins were extracted and used. Only one band at 33 KDa appeared in extracts of all three PC cell lines. **f**. Immunofluorescence assay with homemade EN2 monoclonal antibody. From left to right: the image “ EN2-RFP “ was 293 T cells transfected with EN2-RFP, the image “anti-EN2-FITC” was 293 T cells stained with homemade EN2 monoclonal antibody, the image “isotype antibody” was 293 T cells stained with an control antibody, the image “merge” was merged image of “EN2-RFP “and “anti-EN2-FITC” or “isotype antibody”. Zoom in× 400. EN2-RFP fusion protein gave off red fluorescence, EN2 monoclonal antibody gave off green fluorescence since FITC was labeled at the second detection antibodies, the merged region in “merge” turned yellow in color. Full-length gels and blots are presented in Supplementary Figure 1,2 and 3. These experiments were repeated independently 3 times with similar results

The crystal diagram of EN2 protein was shown in Fig. 1a. The helix 3 region of EN2 protein was expressed and detected by SDS-PAGE (Fig. 1b). The band of EN2 Helix 3, whose size was about 25 KDa, was shown in Fig. 1b. The total protein of 293 T cells transfected with or without EN2-RFP-expressing plasmid was shown in Fig. 1c, marked with “EN2-RFP+” and “EN2-RFP-”, respectively. The band indicated by the red arrow in the lane of “EN2-RFP+” was the EN2-RFP fusion protein expressed by transfected 293 T cells which did not appear in the lane of “EN2-RFP-”. The endogenous and exogenous EN2 identified by WB using our homemade EN2 monoclonal antibody was shown in Fig. 1d, where two bands appeared in “EN2-RFP+” line while only one band appeared in “EN2-RFP-” line. The band at 40 KDa was exogenous EN2-RFP which has the same size as the one indicated by the red arrow in Fig. 1c. The band at 33 KDa was endogenous EN2 protein in 293 T cells. Band of endogenous EN2 in the “EN2-RFP+” lane was weaker than what in the “EN2-RFP-” lane, suggesting the expression of exogenous EN2 weakened that of endogenous EN2. It could also be seen that in 293 T cells, the expression of endogenous EN2 was quite weak, compared with the exogenous one. To validate the specificity of our homemade EN2 antibody in prostate cancer cell lines, total cell proteins of LNCap, DU145 and PC3 were used to identify the endogenous EN2 expression by WB, shown in Fig. 1e. Only one band at 33 KDa appeared in these three cell lines, with the same size as endogenous EN2 in 293 T.

To further validate the antibody specificity, we used the homemade EN2 monoclonal antibody or its isotype control as the first antibody in immunofluorescence to detect the exogenous EN2-RFP fusion protein expressed by transfected 293 T cells, and used FITC-labeled anti-mouse IgG polyclonal antibody as the second antibody to get the green positive signals. The 293 T cells transfected with EN2-RFP-expressing plasmid turned red in color. The representative images were shown in Fig. 1f. Twelve hours after transfection, strong red fluorescence in the nuclei of transfected 293 T cells could be observed through a fluorescence microscope, while no strong red fluorescence was observed on cytomembrane or in cytoplasma. Immunofluorescence results also showed the green signals resulted from EN2 –EN2 antibody-IgG-FITC complex existed mainly in the cell nuclei. The green signals in immunofluorescence merged well with the red fluorescence from EN2-RFP. As a negative control, the images stained with isotype antibody have no green signals since the isotype antibody could not bind the EN2-RFP transfected protein in 293 T cell. All the photos were taken at the magnification of 400 × .

### Subcellular localization of endogenous and exogenous EN2 in LNcap, DU145 and PC3

**Fig. 2:**
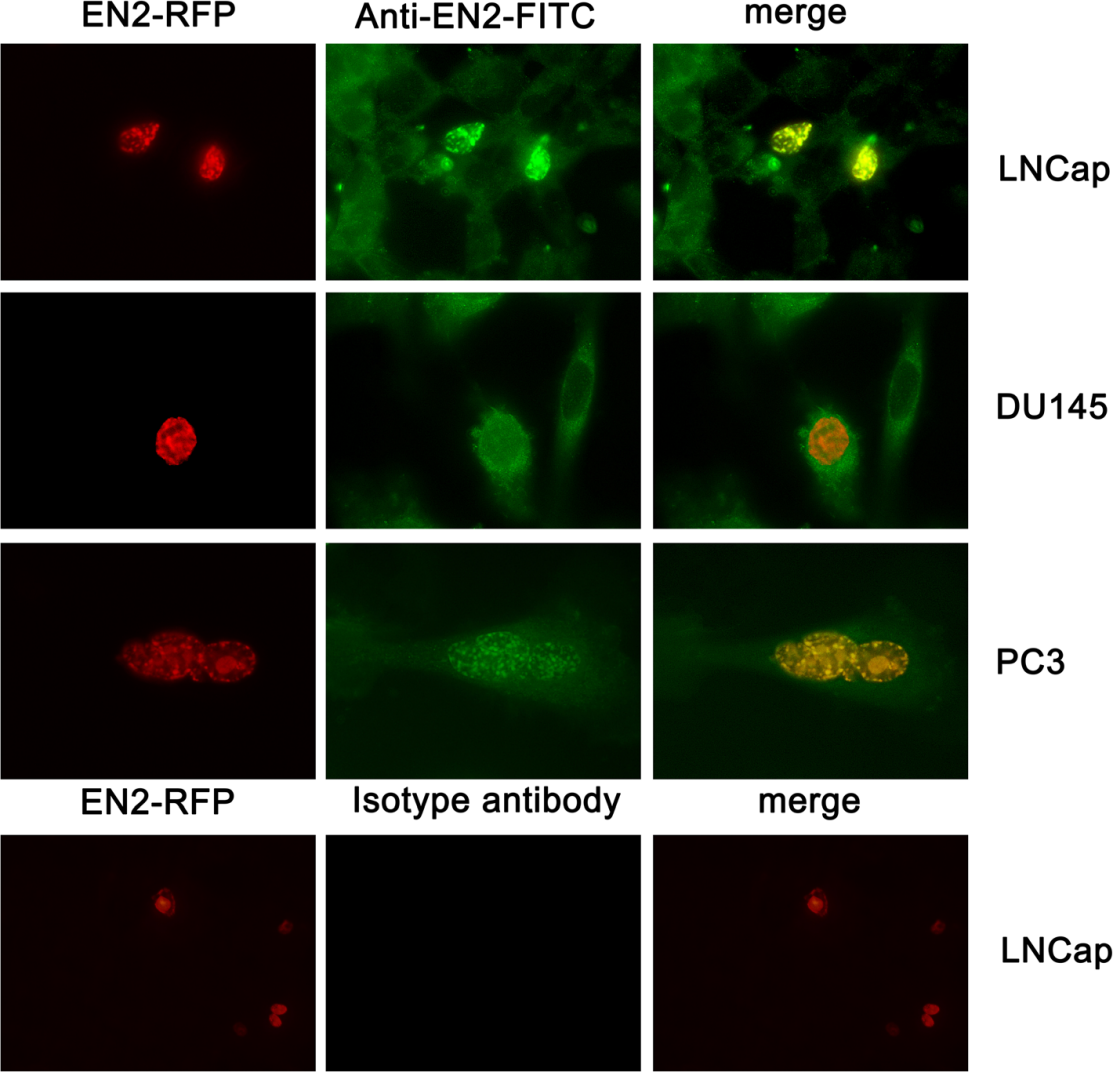
Subcellular localization of exogenous and endogenous EN2 proteins in three PC cell lines. From top to bottom were LNCap, DU145, PC3 and LNCap cell lines. From left to right: the images “EN2-RFP” were LNCap, DU145, PC3 and LNCap cell lines transfected with EN2-RFP, the images “Anti-EN2-FITC” were LNCap, DU145, PC3 cell lines stained with homemade EN2 monoclonal antibody, the image “isotype antibody” was LNCap cell line stained with an isotype antibody as a negative control, the images “merge” were merged images of “ EN2-RFP “ and “Anti-EN2-FITC” or “isotype antibody”. In the left panel, exogenous EN2-RFP fusion protein gave off red fluorescence. In the middle panel, EN2 recognized by the monoclonal antibody gave off green fluorescence while no green fluorescence was detected when isotype antibody used as a negative control. Exogenous EN2-RFP fusion protein showed bright green while endogenous EN2 protein showed weak green stained with EN2 monoclonal antibody. Exogenous EN2-RFP fusion protein distributed in nucleus while the nucleus without exogenous EN2-RFP fusion protein showed as dark hole. The right panel was the merged images of left and middle panel. The sites with yellow color was the overlay of bright green and red color. The magnification of all image was 1000×. These experiments were repeated independently 3 times with similar results

To detect the subcellular localization of endogenous and exogenous EN2 in different types of PC cell lines, LNCap, DU145 and PC3 cell lines which represented different stages of PC were transfected with EN2-RFP-expressing plasmid and then detected by immunofluorescence using our homemade EN2 monoclonal antibody or its isotype control antibody. Twelve hours after transfection, red fluorescence which indicated the exogenous EN2 could be observed through a fluorescence microscope. Then the cells were fixed and immunostained with our homemade antibody or its isotype control, the FITC labeled second antibody against EN2 monoclonal antibody could indicate all EN2 protein, both endogenous and exogenous EN2, in these cells. The representative images were shown in Fig. 2. All the photos were taken at the magnification of 1000 × .

As shown in the left panel of Fig. 2, the exogenous EN2 with red color only distributed in the nuclei of all three cell lines, and there was almost no exogenous EN2 existed in the cytoplasm. As shown in the middle panel of Fig. 2, endogenous EN2 stained with grainy green flurescence distributed in the cytoplasm of three PC cell lines uniformly. And the strong green staining indicating the exogenous EN2 was observed in the nuclei of these three PC cell lines. In those cells not successfully transfected with exogenous EN2, a dark and round nucleus outlined in the cells with weak green signals distributed in the cytoplasm. While in the cells expressing exogenous EN2, the green color was much heavier in nucleus than in cytoplasm. The merged images were shown in the right penal of Fig. 2, indicating only successfully transfected cells had strong yellow staining merged in the nucleus. Other cells without EN2-RFP transfection showed no sign of color in the nucleus, there was the only dark image in the nucleus. The results demonstrated different expression patterns of endogenous and exogenous EN2 in PC cells. As negative controls, images stained with isotype antibody showed no green signal.

### PC tissues have generally stronger EN2 staining on cytomembrane than BPH tissues

To detect the expression patterns of EN2 in PC and BPH tissues, we performed immunohistochemical staining of a series of paraffin-embedded slices from human prostatic samples collected as previously described, and evaluated the staining results by 2 independent pathologists who were both blind to the groups. Carcinoma tissue was confirmed in the PC samples and no carcinoma tissue was confirmed in the BPH samples by the pathologists and EN2 was mainly expressed in glandular and/or carcinoma cells. Table 2 summarized EN2 immunohistochemical scores of BPH and PC. 48% (12/25) of BPH tissues and 100% (25/25) of PC tissues showed EN2 positive staining. Among them, 12% (3/25) of BPH tissues and 72% (18/25) of PC tissues showed EN2 strong staining as well. 52% (13/25) of BPH tissues and 0% (0/25) of PC tissues showed EN2 negative staining. Fisher’s Exact Test showed that the expression level of EN2 in PC and BPH was significantly different, and the expression level of EN2 was much higher in PC group than that in BPH group (*P* < 0.001).

**Table 2:** EN2 immunohistochemical scores of BPH and PC

	BPH (*n* = 25)	PC (*n* = 25)	P
Negative	13 (52%)	0	<0.001*
Positive	12 (48%)	25	
Mild	5 (20%)	3 (12%)	<0.001*
Moderate	4 (16%)	4 (16%)	
Strong	3 (12%)	18 (72%)	

*Fisher’s Exact Test

**Fig. 3:**
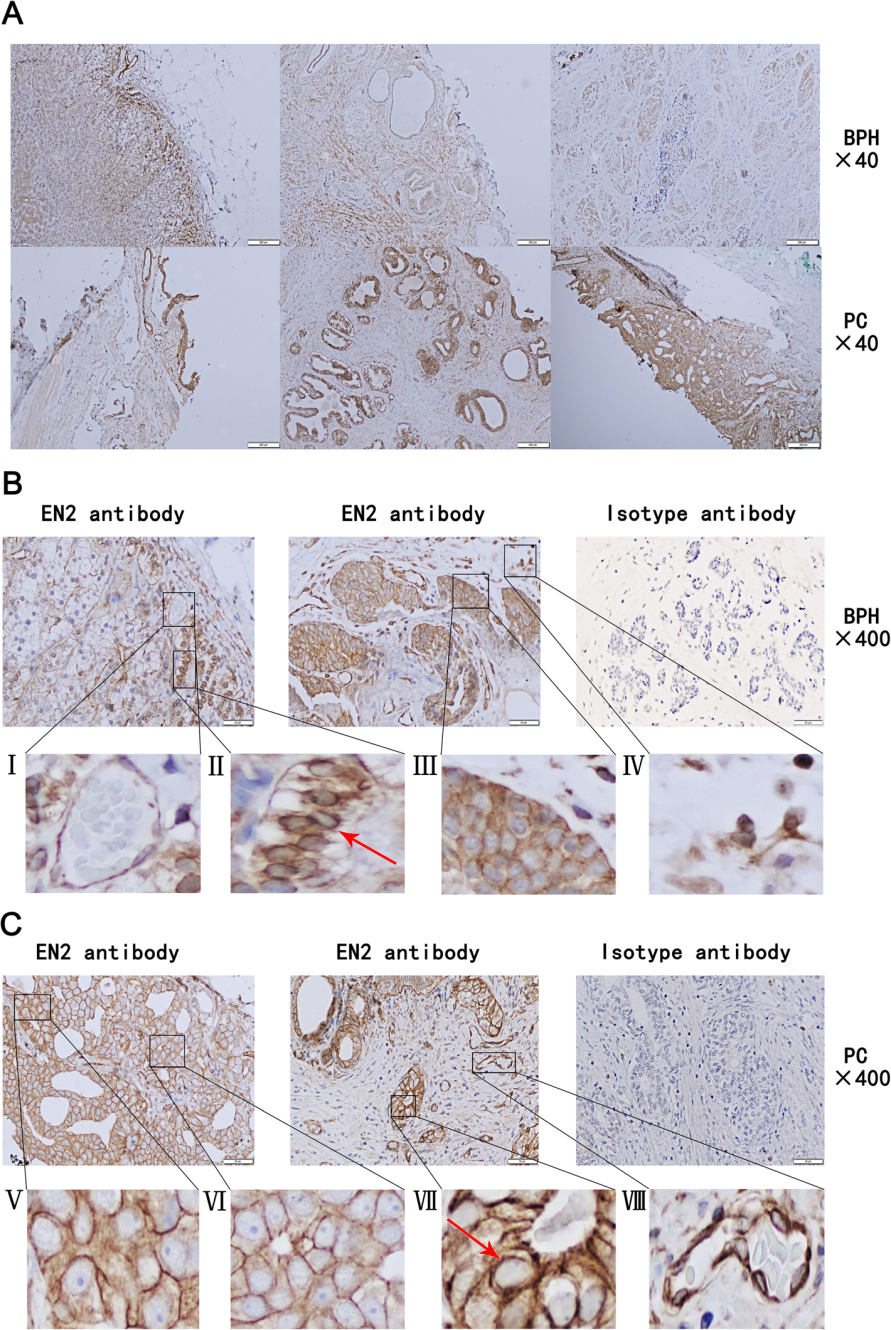
Representative EN2 immunohistochemical images of PC and BPH. **a**. Representative PC and BPH slices. The upper 3 were BPH slices, the lower 3 were PC slices. Zoom in × 40. **b**. Two representative BPH slices with strong staining of EN2 and one negative BPH slice with isotype antibody. The staining patterns of EN2 in the left panel was mainly cytomembrane staining, with clear boundaries and sharp contours among cells. There was shallow staining inside the cells, but nuclear membrane of glandular epithelial cells in the basal part of the prostate gland was obviously stained. “I” was neovascularization endothelial membrane stained with EN2 antibody. “II” was glandular epithelial membranes, nucleus and nuclear membrane (indicated by the red arrow) stained with EN2 antibody. In the middle panel, strong staining in glands was shown. “III” was glandular epithelial cytoplasm stained with EN2 antibody. “IV” was nuclear staining in interstitial tissue. The positive staining cells with round nucleus confirmed by the HE staining were infiltrating lymphocytes. In the right panel, negative BPH slice stained with an isotype antibody. **c**. Two representative PC slices stained with EN2 antibody and one negative PC slice stained with isotype antibody. The EN2 staining sites were mainly focused on the glandular epithelial membrane, glandular cells were well-defined honeycomb-like. “V” and “VI” were cytomembrane staining of EN2 on glandular epithelium cells. “VII” was nuclear membrane staining of EN2 on glandular epithelium cells (indicated by the red arrow). “VIII” was EN2 staining on tumor neovascularization endothelial cells

**Fig. 4:**
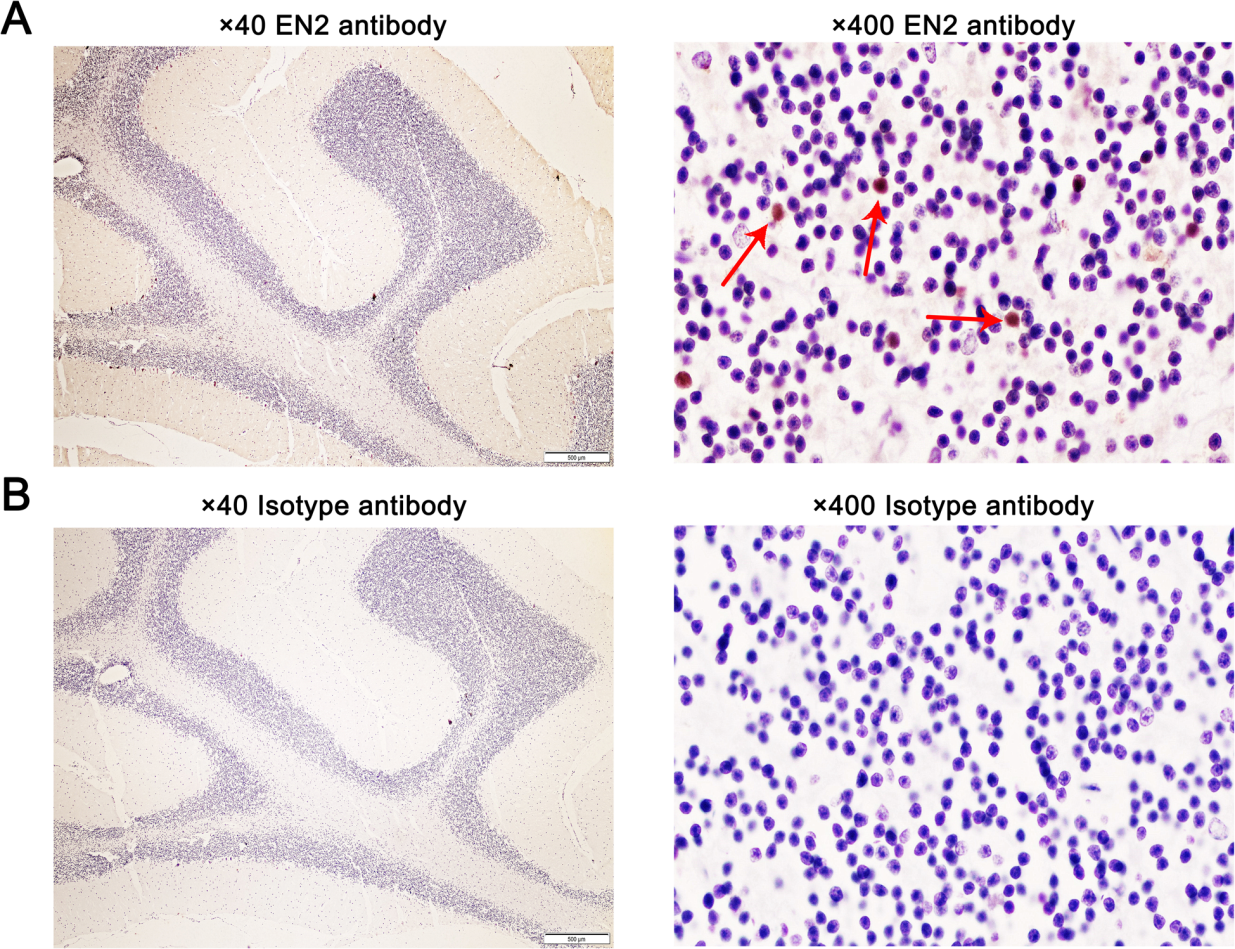
Nucleus expression of EN2 in cerebellar tissues. **a** Representative cerebellar slice stained with EN2 antibody. **b** Representative cerebellar slice stained with isotype antibody as a negative control. Left panel was magnified at 40×. Right panel was magnified at 400×. Positive nucleus staining was indicated by the red arrow

The representative immunohistochemical images of BPH and PC were shown in Fig. 3a. Three images above were BPH slices and the three images below were PC slices. The photos were taken at the magnification of 40×. The staining intensity was weaker in BPH slices than in PC slices. Two strong positive BPH slices stained with EN2 antibody and one stained with negative isotype control antibody were shown in Fig. 3b and four partial enlargements of the photos were shown in I, II, III and IV. In the left panel of Fig. 3b, EN2 was strongly stained mostly in neovascularization endothelial cells and glandular epithelial cells, as shown in “I” and“II”, respectively. Strong EN2 staining on nuclear membrane in BPH tissues was indicated by the red arrow. In the middle panel of Fig. 3b, strong EN2 staining on the gland could be observed. Cytoplasm staining of EN2 was shown in “III”, while scattered EN2 staining in the nuclei of lymphocytes infiltrating in interstitial tissues was shown in “IV”. A negative control stained with isotype antibody was shown in the right panel of Fig. 3b. The photos were taken at the magnification of 400×. Two PC slices stained with EN2 antibody and one stained with isotype control antibody were shown in Fig. 3c (at the magnification of 400× as well). Four partial enlargements of the photos were shown in V, VI, VII, and VIII. In the left panel of Fig. 3c, cytomembrane staining of EN2 on the glandular epithelial cells with well-defined honeycomb-like was shown in “V” and “VI”. In the middle panel of Fig. 3C, strong nuclear membrane staining of EN2 was shown in “VII” and EN2 staining on the tumor neovascularization endothelial cells was shown in “VIII”. The negative control stained with isotype antibody was shown in the right panel of Fig. 3c. The photos were taken at the magnification of 400×. Cerebellum is known for its high expression of EN2 and was stained as a positive control. EN2 antibody staining was shown in Fig. 4a and isotype control antibody staining was shown in Fig. 4b. The magnification of left and right panel was 40× and 400× respectively. EN2 staining was apparently observed in the nucleus in cerebellar tissues. The positive staining was indicated by the red arrow.

In summary, EN2 could be stained in both glandular epithelial cells and neovascularization endothelial cells which are both epithelial original. In glandular epithelial cells, EN2 could be stained on cytomembrane and nuclear membrane, as well as in the cytoplasm and nucleus. Strong staining on cytomembrane was always found in PC slices. The results indicated that EN2 expression patterns changed and the expression level increased as the growth of cells. Different states of the EN2 staining patterns, from the nucleus, nuclear membrane, cytoplasm and cytomembrane in BPH to mainly appeared on cytomembrane in PC suggest that EN2 might be secreted out of epithelial cells especially glandular epithelial cells during the malignant transformation of PC cells. Infiltrating lymphocytes in BPH could also be stained with EN2 antibody suggesting that EN2 protein could be expressed or endocytosed by infiltrating lymphocytes.

### High expression of EN2 both in PC and BHP compared to other four biomarkers

**Fig. 5:**
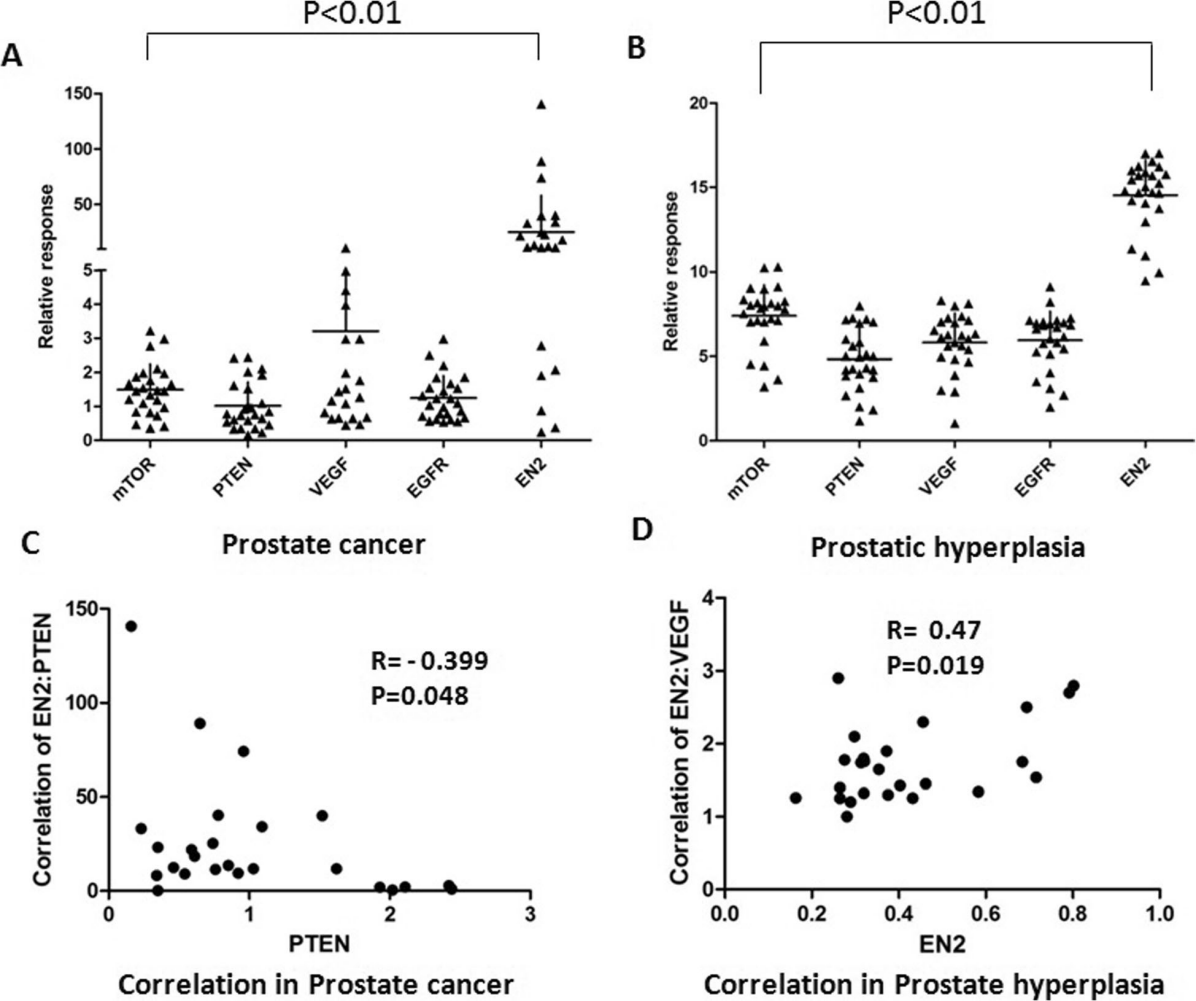
RT-qPCR assay of mTOR, PTEN, VEGF, EGFR and EN2. **a**. Relative transcription levels of mTOR, PTEN, VEGF, EGFR and EN2 in 25 PC cases. **b**. Relative transcription levels of mTOR, PTEN, VEGF, EGFR and EN2 in 25 BPH cases. Each dot represented one case. The mean of relative transcription levels of EN2 was the highest among the mean of relative transcription levels of the other four targets both in PC and BPH cases (*P* < 0.01). **c**. The negative correlation between PTEN and EN2 in 25 PC cases (R = -0.399, *P* = 0.048). No significant difference among other tested markers. **d**. The positive correlation between VEGF and EN2 in 25 BPH cases (R = -0.47, *P* = 0.019). No significant difference among other tested markers

To further confirm the overexpression of EN2 in PC, we detected the expression of four well-studied biomarker proteins, mTOR (mechanistic target of rapamycin kinase), VEGF (vascular endothelial growth factor), EGFR (epidermal growth factor receptor) and PTEN(gene of phosphate and tension homology deleted on chromsome ten), together with EN2 in these 25 PC and 25 BPH tissues, at mRNA level through real-time PCR. Transcription levels of glyceraldehyde phosphate dehydrogenase (GAPDH) were used as the internal quantitative control for those five targets in BPH tissues and transcription levels of these five targets in BPH tissues were used as control in PC tissues. Three duplicated wells of each target gene were set and three independent tests were done in this study. The results were summarized in Fig. 5a and b, and relative transcription levels of those target genes in 25 cases were represented as dots separately. The relative EN2 expression in 25 PC and 25 BPH tissues was the highest compared to other 4 targets (*P* < 0.01). Also the transcription of EN2 was higher in PC tissues than in BPH tissues, and the transcription level of EN2 in 25 PC tissues had the largest variation. As shown in Fig. 5a, in PC tissues, the highest relative expression of EN2 was above 140 while the lowest was less than 1. Because the control of PC tissues was BPH tissues, 1 stands for the transcription level of EN2 in PC tissues was same as the level in BPH tissues. This result indicated other indexes should be added to make definite diagnosis when the expression level of EN2 is low. All these PC cases in our study were clinically diagnosed, and about 3/25 (12%) cases with low EN2 expression (shown in Table 2) should be re-considered to avoid excessive medical treatment, since all these 3 PC patients with low EN2 expression had no lymph node metastasis and good prognosis after excision. For the BPH patients with high level of EN2, regular review should be required since it had possibility for carcinogenesis.

Correlation analysis was performed among mTOR, VEGF, EGFR, PTEN and EN2 both in PC and BPH tissues. There were a negative correlation only between EN2 and PTEN in 25 PC tissues (*R* = -0.399, *P* = 0.048) (Fig. 5c) and a positive correlation only between EN2 and VEGF in 25 BPH tissues (*R* = 0.47, *P* = 0.019) (Fig. 5d). No significant difference among other tested markers in PC and BPH tissues. Since PTEN is a tumor suppressor protein proven in several tumors and VEGF is an inflammatory cytokine related to hyperplasia and tumor, EN2 has been confirmed to be positively related to the carcinogenesis of prostatic diseases.

### EN2 had positive correlation with PC clinical staging

The results above indicated that EN2 has different expression level and distribution in PC and BPH. To further confirm the relationship between EN2 and the progression of PC, we analyzed the clinical indicators among these cases (Table 3), and the correlation between EN2 immunohistochemical scores and clinical indicators in PC (Table 4). As shown in Table 3, only lymphocyte count between BPH and PC groups was significantly different (*P* = 0.001). Peripheral blood lymphocyte number in PC group was higher than that in BPH group. Through Mann-Whitney U Test, the difference of PSA and EN2 immunohistochemical scores between PC and BPH groups were statistically significant (*P* < 0.0001). The PSA and EN2 immunohistochemical scores in PC group were higher than those in BPH group. As shown in Table 4, there was a positive correlation between EN2 immunohistochemical score and PC clinical staging, with the correlation coefficient of 0.428 and the *P* value of 0.033. And more advanced clinical staging, higher EN2 immunohistochemical score. Clinical staging was based on the AJCC guidelines for prostate cancer.

**Table 3:** Clinical indicators of PC and BPH

Parameters	PC (*n* = 25) Mean ± SD	BPH (*n* = 25) Mean ± SD	t/U	P
Age (years)	67.80 ± 7.41	66.12 ± 5.019	0.939	0.352
Smoking history (%)	10 (40%)	7 (28%)	0.802	0.370**
Drinking history (%)	9 (36%)	7 (28%)	0.368	0.544**
White Blood Cell Count(× 10^9^/L)	6.36 ± 1.93	5.91 ± 1.46	0.930	0.357
Platelets Count(× 10^9^/L)	201.68 ± 67.20	170.60 ± 63.58	1.680	0.099
Neutrophil Count(×10^9^/L)	3.70 ± 1.57	3.66 ± 1.33	−0.068	0.946*
Lymphocyte Count(×10^9^/L)	1.89 ± 0.63	1.18 ± 0.74	3.636	0.001
Monocyte Count(×10^9^/L)	0.54 ± 0.18	0.50 ± 0.19	−0.903	0.367*
PSA (ng/ml)	88.76 ± 97.36	2.90 ± 1.47	−6.066	<0.0001*
Immunohistochemical staining score of EN2	3.34 ± 0.96	1.10 ± 1.39	−4.472	<0.0001*

*Mann-Whitney U Test

**Chi-square Test

**Table 4:** Correlation between EN2 immunohistochemical scores and clinical indicators in PC

Clinical indicators	r	P
PC clinical stage	0.428	0.033
Gleason	0.040	0.849
PSA	0.108	0.606
Age	−0.148	0.479
Smoking history	0.238	0.252
Drinking history	0.241	0.246
White Blood Cell Count	−0.230	0.268
Platelets Count	0.022	0.916
Neutrophil Count	−0.282	0.172
Lymphocyte Count	−0.015	0.942
Monocyte Count	−0.028	0.895

**Fig. 6:**
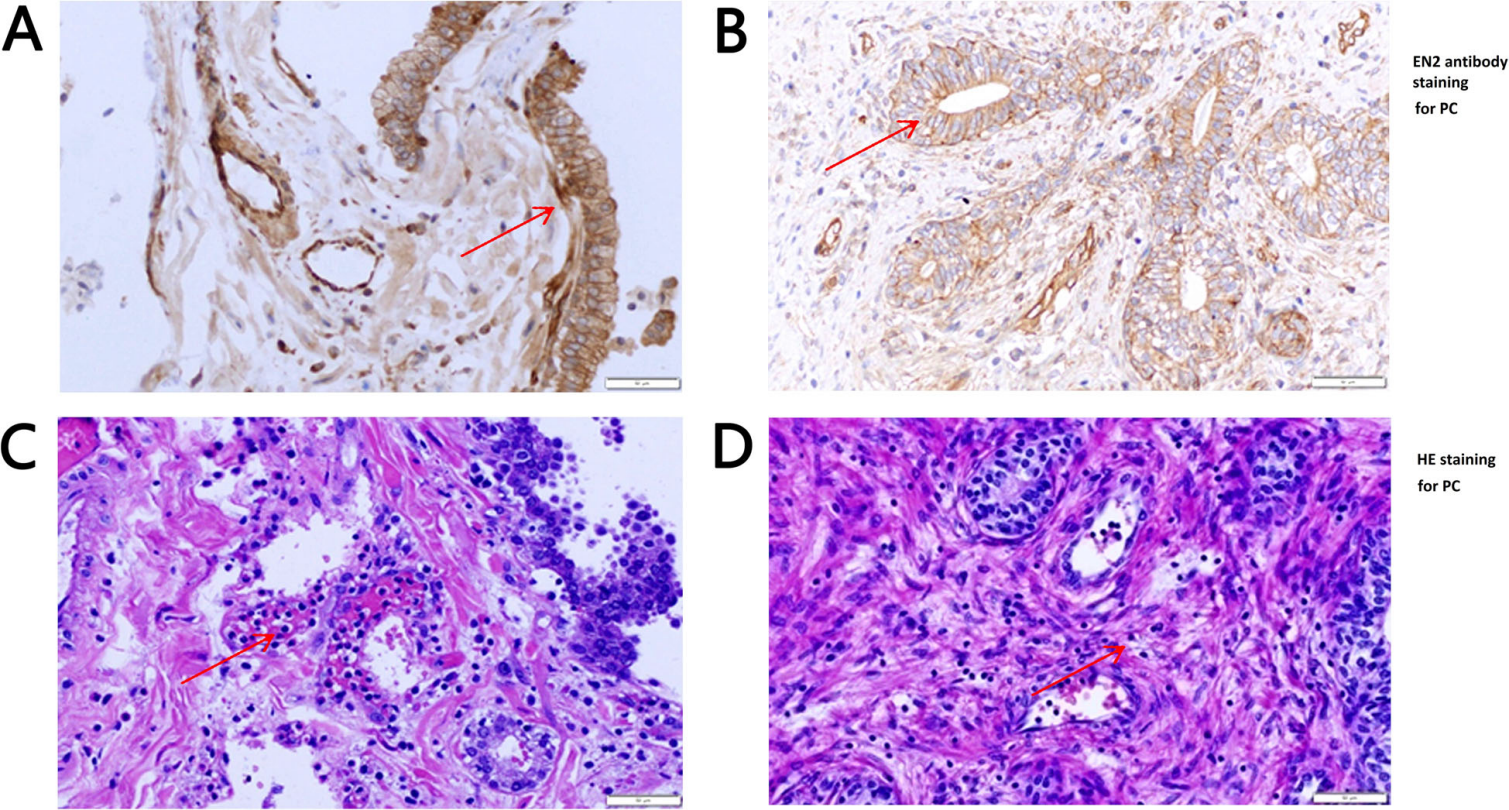
EN2 expression and immune cell infiltration in two PC cases. **a**. Strong EN2 staining in PC slice. There were strong staining in linear boundaries of basilar and lumen sides (indicated by the red arrow). Gland structure was heterogeneous. **b**. Moderate EN2 staining in PC slice. There were strong EN2 staining in lumen sides. EN2 distribution in lumen sides showed ascending form with obvious polarized distribution (indicated by the red arrow). **c** and **d** were HE staining of same slices corresponding to A and B. There were numerous neutrophil infiltration (shown in C) and lymphocytes infiltration (shown in D). Neutrophils mainly distributed in the blood vessels, while lymphocytes mainly distributed in the interstitial indicated by red arrow

EN2 was correlated with clinical stage could be proven from one sight. In this study, neutrophil or lymphocyte infiltration were found in some cases, where the EN2 expression also could be detected. One patient with neutrophil infiltration was at clinical stage IV, and one patient with lymphocyte infiltration was at clinical stage II. The distribution, morphology and expression level of EN2 were also different in these two cases. As shown in the previous studies, prognosis of tumor tissues infiltrated by neutrophil was poor, while that of tumor tissues infiltrated by lymphocytes was good [14, 15]. In Fig. 6a, high expression level of EN2 and cell heteromorphosis were indicated by the red arrows. Numerous lobulated neutrophils in capillaries (indicated by the red arrow) could be observed in Fig. 6c, the same tissues as in Fig. 6a but were stained with HE. The PC patient at clinical stage IV was relapsed 1 month after resection. The expression level of EN2 was low In another case shown in Fig. 6b, whose glandular morphology was intact and EN2 polarly distributed on the edge of the glandular cells, indicated by the red arrow. A large amount of lymphocyte infiltration could be observed (indicated by the red arrow) in Fig. 6d, the same tissue as in Fig. 6b were stained with HE. This PC patient at clinical stage II had never relapsed in 1 year since recovery and never been subjected to hormonotherapy.

## Discussion

BPH and PC are progressive diseases [16]. Accurate diagnosis can not only improve the cancer treatment but also avoid clinical overtreatment. EN2 had been well studied in the field of neurodevelopment. More and more studies were shown its potential association with tumorigenesis. In this study, we found that EN2 expression pattern and level changed as the prostatic disease progresses. Continuous monitoring of EN2 might be a helpful method for prognosis judgment.

The EN2 Helix 3 has been confirmed to be the main functional structural domain of the protein, mediating its exocrine and internalization [17, 18]. Some studies have reported that antibodies against this domain of EN2 could identify the concentration of EN2 in PC patients’ urine [10, 11]. In this study, we used monoclonal antibody against EN2 Helix 3 could distinguish the distribution difference between PC and BPH. We found EN2 mostly localized in the nuclei of cerebellar tissue. Interestingly, EN2 in PC mainly expressed on cytomembrane or expressed as an exocrine expression, while EN2 in BPH mostly expressed in cytoplasm and nuclei. Furthermore, the expression level of EN2 in PC was higher than that in BPH. This interesting finding can help doctors to judge the progress of prostatic diseases and help scientists to understand the characteristics of EN2 in different tissues. However, the precise criteria of EN2 to define PC or BPH can’t been given from this study because of the limited samples.

It is noteworthy that the subcellular distribution of EN2 in PC cell lines and PC tissues were not exactly the same. Cytoplasm staining pattern was observed in all three PC cell lines which was usually seen in BPH tissues. Weak nucleus staining pattern in all three PC cell lines was usually in PC tissues. But strong cytomembrane staining pattern was only observed in prostatic malignant tissues. EN2 itself is a transcription factor that interacts with DNA and is supposed to exist in the nucleus for normal cells. Because the expression and characteristic of EN2 could change due to the change of cell proliferation, EN2 could be secreted into the cytoplasm and even extracellular in cancer cells [13, 19]. Some studies have reported that LNCap, DU145 and PC3 cells represent three different stages of prostate cancer [20–22], but in this study, no significant differences of EN2 in the subcellular localization was found among these three cell lines. It is possible that the number of passages and the artificial medium conditions in vitro led to changes in the original tumor malignancy of the cell lines.

Limited to the sample number, no statistical difference between any two clinical indexes was found, except for significant difference between EN2 and the clinical staging of PC. This result further confirmed the difference of EN2 expression level and patterns between BPH and PC could suggest the progress and prognosis of prostatic diseases. Studies with more clinical samples are needed to confirm our new finding and set up a precise criteria for using EN2 as a biomarker in prostatic diseases.

## Conclusions

HOX family plays a key role in cell proliferation. EN2, one member in HOX family, was found to have different expression levels and patterns in PC and BPH, and its expression level was positively correlated with the PC clinical staging, suggesting its use to predict prostatic disease progression.

## Supplementary information

**Additional file 1.** Figure S1.

**Additional file 2.** Figure S2.

**Additional file 3.** Figure S3.

## Data Availability

The authors declare that they have no competing interests.

## Abbreviations

EN2: Engrailed-2
PSA: Prostate-specific antigen
PC: Prostate cancer
BPH: Benign prostatic hyperplasia
RT-qPCR: Reverse Transcription quantitative Polymerase Chain Reaction
WB: Western Blotting
ELISA: Enzyme-linked immunosorbent assay
LUTS: Lower urinary tract symptoms
HOX: Homeobox
DNA: DeoxyriboNucleic Acid
RFP: Red fluorescent protein
PEI: Polyethylenimine linear
DMEM: Gibco Dulbecco’s Modified Eagle Medium
PBS: Phosphate buffer saline
BSA: Bovine serum albumin
FITC: Fluorescein isothiocyanate
EDTA: Ethylene Diamine Tetraacetic Acid
DAB: Diaminobenzidine
HE: Hematoxylin-eosin
GAPDH: Glyceraldehyde-3-phosphate dehydrogenase
SDS-PAGE: Dodecyl sulfate,sodium salt-Polyacrylamide gel electrophoresis
mTOR: Mechanistic target of rapamycin kinase
VEGF: Vascular endothelial growth factor
EGFR: Epidermal growth factor receptor
PTEN: Gene of phosphate and tension homology deleted on chromsome ten

## References

[1] Grozescu T (2017). Prostate cancer between prognosis and adequate/proper therapy. J Med Life.

[2] Barry MJ (2017). Prevention of prostate Cancer morbidity and mortality: primary prevention and early detection. Med Clin North Am.

[3] Sebesta EM (2017). The surgical Management of Prostate Cancer. Semin Oncol.

[4] Wallis CJD (2016). Surgery versus radiotherapy for clinically-localized prostate Cancer: a systematic review and meta-analysis. Eur Urol.

[5] Andriole GL (2009). Mortality results from a randomized prostate-cancer screening trial. N Engl J Med.

[6] Dai X (2016). Benign prostatic hyperplasia and the risk of prostate Cancer and bladder Cancer: a meta-analysis of observational studies. Medicine (Baltimore).

[7] Mokhtari M (2016). The prevalence of prostatic stromal tumor of uncertain malignant potential in specimens diagnosed as prostatic hyperplasia. Arch Iran Med.

[8] Morgan R (2017). Targeting HOX/PBX dimers in cancer. Oncotarget.

[9] Lai CY (2016). Engrailed-2 might play an anti-oncogenic role in clear-cell renal cell carcinoma. J Mol Histol.

[10] McGrath SE (2015). EN2 in prostate Cancer. Adv Clin Chem.

[11] Pandha H (2012). Urinary engrailed-2 (EN2) levels predict tumour volume in men undergoing radical prostatectomy for prostate cancer. BJU Int.

[12] Carlier L (2013). Investigation of homeodomain membrane translocation properties: insights from the structure determination of engrailed-2 homeodomain in aqueous and membrane-mimetic environments. Biophys J.

[13] Natasha P (2019). Membrane insertion and secretion of the Engrailed-2 (EN2) transcription factor by prostate cancer cells may induce antiviral activity in the stroma. Sci Rep.

[14] Watanabe A (2019). Absolute neutrophil count predicts postoperative prognosis in mass-forming intrahepatic Cholangiocarcinoma. Anticancer Res.

[15] Kim J (2016). Tumor-associated macrophages and neutrophils in tumor microenvironment. Mediat Inflamm.

[16] Donnel RF (2011). Benign prostate hyperplasia: a review of the year's progress from bench to clinic. Curr Opin Urol.

[17] Joliot A (1998). Identification of a signal sequence necessary for the unconventional secretion of engrailed homeoprotein. CurrBiol.

[18] Logan C (1992). Cloning and sequence comparison of the mouse, human, and chicken engrailed genes reveal potential functional domains and regulatory regions. Dev Genet.

[19] McGrath SE (2018). Engrailed-2 (EN2) - a novel biomarker in epithelial ovarian cancer. BMC Cancer.

[20] Scaccianoce E (2003). Characterization of Prostate Cancer DU145 Cells Expressing the Recombinant Androgen Receptor. Oncol Res.

[21] Altuwaijri S (2007). Expression of human AR cDNA driven by its own promoter results in mild promotion, but not suppression, of growth in human prostate cancer PC-3 cells. Asian J Androl.

[22] Li J, et al. SHARPIN overexpression induces tumorigenesis in human prostate cancer LNCaP, DU145 and PC-3 cells via NF-jB/ERK/Akt signaling pathway. Med Oncol. 2015;32(2):444. 10.1007/s12032-014-0444-3 25550157

