# Supplementary figures and images for: Altered staining patterns and expression level of Engrailed-2 in benign prostatic hyperplasia and prostate Cancer predict prostatic disease progression
Source: BMC Cancer. 2020 Jun 15;20:555. doi: 10.1186/s12885-020-07049-z (PMC7296936; doi:10.1186/s12885-020-07049-z)

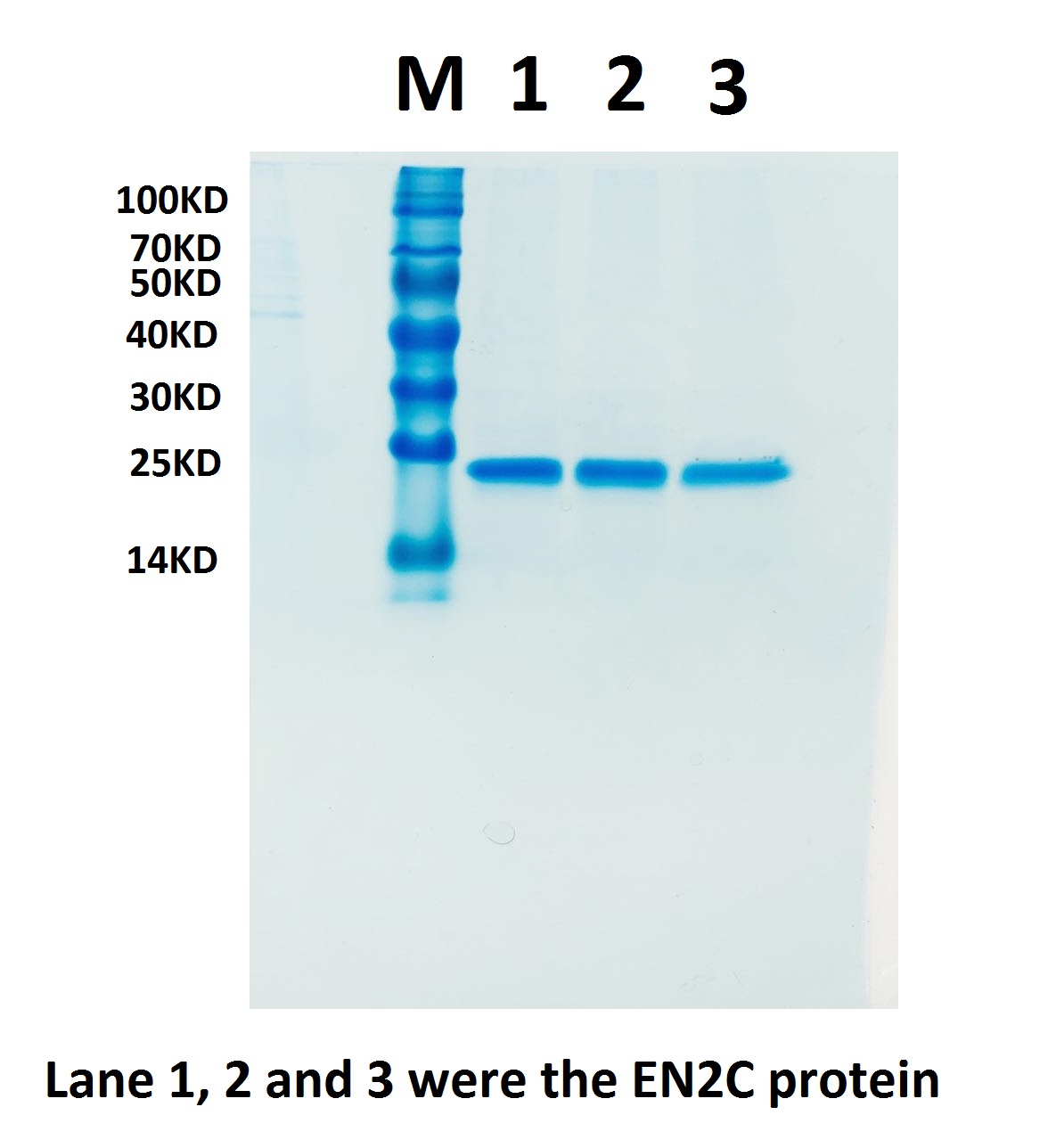

Supplement: Supplementary file 1 — Additional file 1. Figure S1. [file 12885_2020_7049_MOESM1_ESM.jpg]

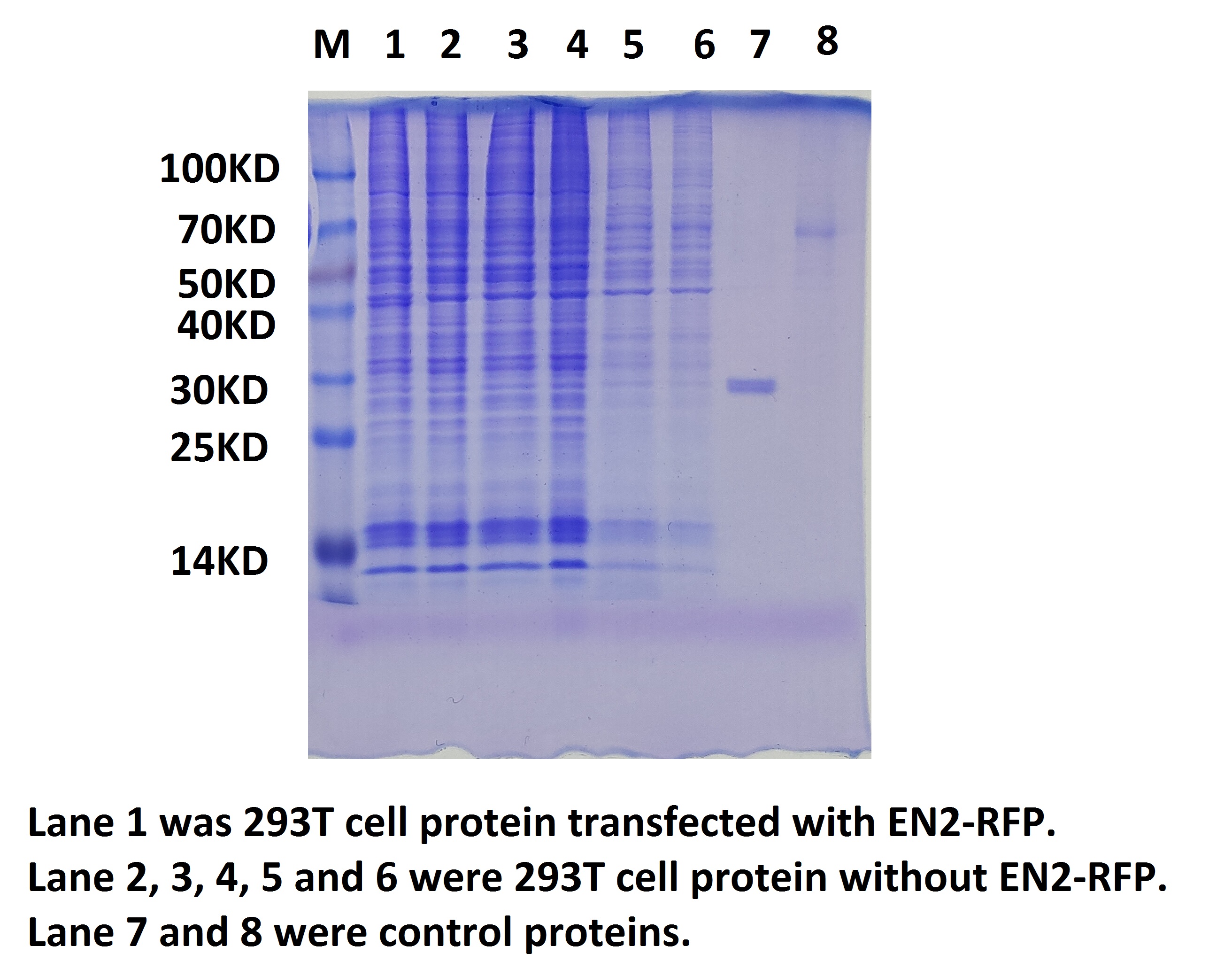

Supplement: Supplementary file 2 — Additional file 2. Figure S2. [file 12885_2020_7049_MOESM2_ESM.jpg]

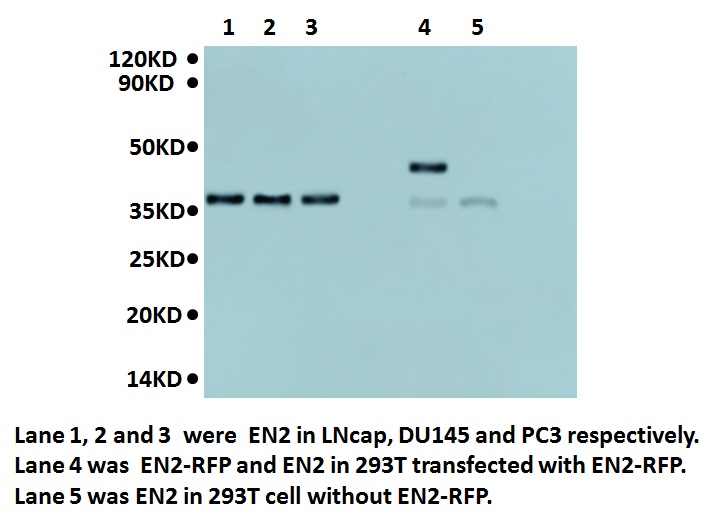

Supplement: Supplementary file 3 — Additional file 3. Figure S3. [file 12885_2020_7049_MOESM3_ESM.jpg]
